# Development and validation of the ADHD Symptom and Side Effect Tracking - Baseline Scale (ASSET-BS): a novel short screening measure for ADHD in clinical populations

**DOI:** 10.1186/s12888-023-05295-6

**Published:** 2023-11-06

**Authors:** Joel L. Young, Richard N. Powell, Celeste Zabel, Jaime Saal, Lisa L. M. Welling, Jillian Fortain, Ashley Ceresnie

**Affiliations:** 1 Rochester Center for Behavioral Medicine, 441 South Livernois, Suite 100, Rochester Hills, MI 48307 USA; 2 School of Medicine - Wayne State University, Detroit, USA; 3 Henry Ford Health System, Detroit, USA; 4 Department of Psychology, Oakland University, Rochester, USA

**Keywords:** Adult ADHD, Assessment, Decision making

## Abstract

**Objective:**

The aim was to develop and test a novel screen of adult ADHD, with a specific focus on clinical use. We designed a series of three studies to accomplish this aim.

**Method:**

Study One (*n* = 155) and Study Two (*n* = 591) collected data via surveys to conduct exploratory and confirmatory factor analyses, respectively. Study Three analyzed the scale’s psychometrics in a clinical sample (*n* = 151).

**Results:**

Study One and Study Two identified a 10-item scale with a two-factor structure. Study Three found good discriminant validity, sensitivity = 80.0%, specificity = 80.2%, and convergent validity with both the Brown Executive Function/Attention Scales, *r* (131) = .76, *p* < .001, and the Conner’s Adult ADHD Rating Scales *r* (131) = .71, *p* < .001.

**Conclusion:**

The scale demonstrated effectiveness in screening for ADHD in a psychiatric outpatient population. Its results may be used to identify patients that may benefit from thorough ADHD diagnostic procedures.

**Supplementary Information:**

The online version contains supplementary material available at 10.1186/s12888-023-05295-6.

Attention Deficit Hyperactivity Disorder (ADHD) emerges in childhood and may continue throughout adulthood [1]. Across the lifespan, child and young-to-middle aged adult populations with ADHD suffer impairments in their work, personal, social, and/or school life [2]. Although ADHD creates significant and limiting problems for those afflicted, ADHD generally responds favorably to psychiatric treatment; treated ADHD populations have vastly improved lifetime outcomes compared with untreated ADHD populations [3]. Thus, accurate and timely diagnosis is pivotal when ADHD is present.

Arriving at an accurate ADHD diagnosis poses challenges for psychiatric treatment providers. First, ADHD symptoms may be easily attributable to other mental health disorders [4]. For example, symptoms or signs of ADHD usually include failure to pay attention, forgetting to do everyday tasks, difficulty following instructions or completing a task, trouble organizing, losing important or needed items, talking too much, restlessness, difficulty waiting, and lack of productivity [5]. These signs and symptoms may also be a part of a clinical presentation when other behavioral disorders are the cause, such as oppositional defiant disorder, conduct disorder, and major depressive disorders [6]. Comorbid bipolar disorder and ADHD may have some shared clinical characteristics, especially signs and symptoms of hyperactivity and impulsivity; however, comorbid bipolar disorder and ADHD may also have some distinct clinical characteristics, including an earlier onset of bipolar disorder compared to ADHD [7]. Second, delayed treatment may cause or exacerbate symptoms that might obscure an underlying ADHD diagnosis. For example, mood instability can be a result of untreated ADHD, which may lead a clinician to prescribe mood stabilizing medication instead of attention and behavior regulation-enhancing medication [8].

Diagnosing ADHD is further complicated by every patient’s unique psychological and social developmental history. Across age groups, the measured prevalence of ADHD in adults indicates that men are more likely than women to have ADHD; however, this finding may be inaccurate due to pervasive societal norms and expectations pertaining to gender. In general, girls, more often than boys, are coached by teachers and parents to practice social-emotional regulation skills and not engage in externalizing behaviors [9]. Thus, ADHD in women may be misdiagnosed due to failure to account for how lived experience in relation to gender roles, norms, and expectations may impact observed symptoms. Besides gender, a multitude of other important biopsychosocial variables (i.e. social-economic status, nutrition, history of neglect or abuse, race, comorbid physical and mental health conditions) may have similar modifying effects on how ADHD is presented by each patient [10]. Therefore, evaluating a patient for the possible presence of ADHD requires a thorough clinical conceptualization of each patient developed through information collection via a variety of means and sources.

## ADHD assessment measures

To adapt to challenges caused by symptom presentation and varied biopsychosocial impacts on ADHD presentation, clinicians often use validated psychological assessments in addition to clinical interviews and observations in order to construct an accurate clinical conceptualization of a patient’s presenting symptoms. There are multiple ADHD rating scales used to identify and measure the symptoms of ADHD. However, clinicians face a need for easily accessible short rating scales created for use in identifying adult ADHD within a clinical population. These assessments differ in terms of construct operationalizations (e.g., subjective severity of deficits in executive function, how frequently symptoms occur, degree of symptom interference with functioning, or presence of symptoms across the lifespan); how results are appropriately interpreted (e.g., norm referenced, criterion referenced, or overall likelihood of having an ADHD diagnosis), the perspective of the person rating ADHD (e.g., self-report, observer, educator, or clinician), and for what types of psychiatric assessments they are most appropriately used (e.g., used during the initial patient contact to identify possible diagnostic explanations for the patient’s presentation; or, used as part of an in-depth analysis following the initial contact to test a possible diagnosis).

The current availability of multiple ADHD assessment rating scales, which offer a wide range of valid approaches and operationalizations of ADHD symptoms, benefits clinical practice. A well-selected set of independent ADHD assessment scales increases the likelihood that an ADHD assessment will result in an accurate conceptualization of an individual patient’s unique presentation and reduces risks of either a type I or type II error compared to the use of just one scale [11]. A lack of independence between measures, or reliance on just one measure, may result in deficits in treatment planning if clinician or patient deficits in communication skill, communication-inhibiting interpersonal dynamics between clinician and patient, or lack of rigor in clinical information gathering prevents a clinician from developing a sufficiently complete conceptualization of a patient’s presentation to adequately create a treatment plan. However, a well-selected set of independent ADHD scales would more logically provide the necessary structure and multidimensional investigation of a patient to more fully capture the clinical conceptualization for developing an effective treatment plan. The field’s ability to employ such a comprehensive ADHD assessment is due to having a multitude of valid and reliable, but varied in assessment theory and strategy, measures to select from when evaluating patients who achieved positive screens for ADHD during an initial patient contact.

Yet, the set of available ADHD measures that would be practicable for wide administration to screen patients at initial clinical contact is limited. The Adult Attention Deficit/Hyperactivity Disorder Self-Report Scale (ASRS V1.1) was developed for use by the general public by the World Health Organization and is widely used for initial screening purposes [12]. An updated version of the ASRS’ scoring was found by Ustun et al. [13] to achieve very strong sensitivity (0.91) and acceptable specificity (0.74) in a clinical population. However, the ASRS has mixed results in other published studies evaluating its discriminant validity within clinical populations. Indeed, Van de Glind et al. [14] found overall acceptable discriminant validity when the ASRS was administered on treatment-seeking substance use disorder patients, at least in terms of sensitivity (0.84), but not specificity (0.67); Hines, King and Curry [15] similarly found strong sensitivity (1.0) but weaker specificity (0.71) in a population of primary care patients; and, Dunlop, Wu, and Helms [16] measured low levels of sensitivity (0.60) and specificity (0.69) when used in a population of adults with Major Depressive Disorder. The Conner’s Adult ADHD Rating Scale, brief self-report ADHD Index (CAARS ADHD) assessment is another option [17], but, due to its cost and norm-referenced scoring, the CAARS ADHD Index may be burdensome for many clinicians to employ routinely. If clinicians were able to readily administer a companion short screening scale to the ASRS at patient intake, or have an option to select from more than one short screening scale depending on clinician preference or patient preference, then the ability to identify patients that would be best served by an in depth and comprehensive ADHD assessment would be greatly improved.

Developing a valid and reliable short screening rating scale with a straightforward interpretation, established for use within a psychiatric population, would promote accuracy in ADHD diagnosis and treatment decisions. To this end, here we report on the results of a series of three studies undertaken to develop a brief self-report screening instrument for ADHD, entitled the *ADHD Symptom and Side Effect Tracking - Baseline Scale* (ASSET-BS), for the use of screening for ADHD in a population of patients seeking psychiatric treatment.

## Method

Study One was designed to collect data to perform an exploratory factor analysis (EFA) on proposed items for the new measure to identify a factor structure. Study Two was designed to collect data in a large sample representative of the U.S.A. general population to perform a confirmatory factor analysis of the factor structure identified in Study One. Finally, Study Three was designed to evaluate the ASSET-BS’ performance in terms of convergent validity and discriminant validity in screening for ADHD in a population of patients starting outpatient psychiatric treatment for a wide range of potential DSM-5 problems. To do so, we analyzed ASSET-BS results in the context of a wide-ranging psychiatric assessment battery normally administered prior to initiating outpatient treatment for psychiatric disorders at a large outpatient psychiatric practice.

### Item development

The initial item set was taken from a clinician’s tool developed at a large outpatient psychiatric practice located in the midwestern U.S.A. The clinician’s tool, titled “ADHD Symptom and Side Effect Tracking” (ASSET) was created in 2001, and consisted of a set of DSM-IV criterion-derived items relating to ADHD-related symptoms and common side effects of ADHD pharmacological treatment. The items were changed over time in accordance with DSM-5 changes and as clinicians at the practice requested updates. As of 2019, the ASSET contained 16 items that measured patient-reported change using a scale of -3 (worsening) to +3 (improving).

As the ASSET was purported to be a useful clinical tool to inform treatment decisions and help structure clinical interviews, we hypothesized that if items derived from it were used to measure overall ADHD severity, it would be an ideal short screener for ADHD within a clinical population. Further, a measurement of ADHD symptom severity using a tool that emerged from clinical practice may be uniquely attuned to certain facets of ADHD symptomatology that are responsive to psychiatric treatment.

We determined that 10 of the original 16 items had good criterion agreement with the DSM-5, and 3 of the other 6 items had strong clinical rationale for inclusion as proposed items for a novel ADHD measure. The item *Sleep Quality* was included due to the DSM-5’s reference to the impact of sleep disorders on ADHD symptoms, plus strong cross-cultural evidence reported in peer-reviewed literature that ADHD is highly related to problems with sleeping for both adults and children [18–21]. The item *Anxiety* was included because anxiety may be a moderating factor between childhood ADHD and decreased quality of life in adulthood [22]. Anxiety has also been found to be highly consistent with and related to adult ADHD symptoms and executive functioning deficits [23]. Further, anxiety has been identified as a key clinical feature of the non-inattentive symptoms of ADHD [24]. Therefore, we hypothesized that it would be additive to the measurement model, increasing accuracy. The item *Mood* was included to coincide with clinical reports of the presentation of Adult ADHD. For example, the Mayo Clinic [5] reports that adults with ADHD may experience frustration, mood swings, and problems with temperament. Similarly, children diagnosed with ADHD are found to be angrier and more depressed than children without ADHD [25]. Indeed, the non-inattentive symptoms of ADHD have been linked to and conceptualized as emanating from an overall deficit in managing emotions [24].

The items *Fatigue* and *Muscle/Joint Pain* represent symptoms commonly present at intake for adult ADHD patients [26, 27]. However, they were not included in the proposed baseline scale to maintain criterion validity with the DSM-5 and to limit interference with the myriad of conditions outside of the scope of the DSM-5 associated with fatigue and muscle/joint pain (e.g., diabetes, cancer, and multiple sclerosis). The items *Appetite* and *Dry Mouth* were not included as they primarily reference common side effects of front-line ADHD medications [28]. The clinician-tool version of the ASSET includes a survey of a patient’s self-reported frequency of selected individual side effects. This side effect survey is administered at weekly visits alongside the ADHD symptom questions. These side effect survey questions are reproduced in Supplement 1, but as the side effect survey is not purported to measure underlying constructs, these questions were not included as part of the three separate validation studies reported here. As such, the side effect survey questions reproduced in Supplement 1 are provided as suggestions for clinicians and could be used in future research.

After the initial proposed items were selected, we adopted a 6-point Likert-type scale to measure each item. The scale asks the participant to rate the level of the impact on daily life functioning they may have experienced due to problems with the sign or symptom of ADHD referenced by the item (anchors: 1 = no problem present, 6 = severe impact). We chose to measure the impact of symptoms on daily life functioning to ground the scale in the DSM-5’s diagnostic criteria for clinical significance.

### Procedure and measures

#### Study 1

Participants completed the study via an online survey developed and hosted on a Qualtrics platform. Once individuals consented, the online survey asked participants to identify their gender, race, education level, and if they have ever received a diagnosis of ADHD or have been treated for ADHD. Participants were then directed to answer the 13 initially proposed items for the ASSET-BS.

#### Study 2

The respondent panel selection and survey administration was completed via Qualtrics. Participants were asked to answer the ASSET-BS items compiled in Study One, along with an expanded set of demographic items to confirm stratified sampling targets were reached in terms of race/ethnicity, urban/rural residency, gender, and geographic region of the United States. Two demographic variables were collected but not used as sampling sufficiency targets: educational attainment level, and self-reported history of ADHD diagnosis or treatment.

#### Study 3

The study design was an analysis of an outpatient psychiatric clinical sample’s archived psychological test battery results. The ASSET-BS was included in an assessment battery administered at a large Midwestern United States psychiatric practice. Its results were not used to interpret a patient’s condition. During the assessment, all patients were given the opportunity to decline to answer the ASSET-BS items, even if they had already provided broad consent for research use of their testing data. The wide-range assessment included dozens of possible measures with screeners selected to measure a wide swath of the DSM-5. From the archived assessment battery results, we selected for analysis the Epworth Sleepiness Scale (ESS) [29], the Dimensions of Obsessions and Compulsions Scale (DOCS) [30], and the PHQ-9 [31] to test for divergent validity. We selected the Brown EF/A overall index T-score [32], CAARS Self-Report ADHD Index T-score, the CAARS Self-Report DSM-5 Symptoms Subscale T-score, and the CAARS Observer-Report Index and CAARS Observer-Report DSM 5 Symptoms Subscale T-scores to test for convergent validity [16].

### Participants

#### Study 1

One hundred and seventy participants were recruited through convenience sampling to complete a brief survey comprising the proposed ASSET-BS questions and demographic items. The participants were invited purposefully by the research team either individually or through email through select listservs to select adults who would thoughtfully answer the questions and offer any critiques or suggested changes. To this end, the sample consisted of adult colleagues, coworkers, other psychiatric care providers, graduate students, and classmates of the research team members. Graduate student email listservs were used if members of the research team had pre-existing permission to post research invitations to the listservs [33]. All participants responded at least partially to the survey, and 155 participants (mean age = 32.02, *SD* = 12.22) submitted complete survey responses. Out of those 155 participants, the sample was 83.9% female, 15.5% male, and 0.6% transgender female. Reported race and ethnicity was 83.9% Caucasian, 5.8% African American, and 4.5% Asian. For education background, the sample reported 11.0% doctorate-level degree, 47.7% master-level degree, 17.4% bachelor-level/4-year degree, 19.3% associate-level/two-year degree or some college, and 4.5% reported a high school diploma or had not completed high school. For self-reported ADHD incidence, 26.5% self-reported having been treated for or diagnosed with ADHD as an adult, and 71% indicated that they had not been diagnosed or treated for ADHD as an adult, whereas 2.6% were unsure or declined to disclose whether they had been diagnosed with or treated for ADHD.

#### Study 2

To capture a nationally representative sample, we partnered with Qualtrics to administer a survey to 591 adult participants. The participant set was selected to coincide with U.S. census-reported percentages of ages, gender identities, geographic locations, ethnicity, and race. The sample ranged from 18 to 65 years of age (mean = 39.13, *SD* = 12.54). About 52% of the participants were female, 46.2% were males, and 1.1% of the participants reported a non-cisgender identity such as transgender or non-binary. 65.8% reported being White/Caucasian, 15% Hispanic, 9.5% African American, 3.9% Asian, 2.9% reported being of two or more races, 1.2% American Indian/Alaska Native, and 0.3% endorsed a race/ethnicity of native Hawaiian/other pacific islander. Seventy-five percent of the study population reported living in an urban area of the United States, and 25% reported a rural living location. Education levels varied greatly, with 22.7% of those having graduated high school, 21.3% having a 4-year college degree, about 14% having some college education, about 12% having a professional degree, and 12% also having a graduate, masters level degree. Additionally, a small percentage had either a 2-year college degree or a doctorate. Lastly, 61.6% of the participants reported not having an ADHD diagnosis, whereas 35.2% reported either having an ADHD diagnosis or having received treatment for ADHD, and 3.2% were unsure if they had ever been assigned a diagnosis of ADHD or been treated for ADHD.

#### Study 3

We selected a clinical sample of 151 outpatient adult psychiatric patients (mean age = 30.26, *SD* = 11.38, range = 18 to 71) who had completed a wide-range psychiatric assessment battery within a 2-month timeframe in 2021. To be included in the sample, the assessment battery must have been ordered following an intake session with a clinician, and the patient must have provided broad consent for research use of their psychological testing data. The demographic profile of the resultant clinical sample was as follows: 91.0% were Caucasian, 3.7% Asian, 3.0% African American, and 1.5% self-identified as “other” for race/ethnicity. 64% were female, and 33% were male, and 3% elected to not disclose their gender when taking the psychological assessment battery.

The clinical sample was referred for assessment based on a biopsychosocial evaluation by a clinician that resulted in the clinician submitting a referral form containing hypothesized conditions for which the clinician desired additional information to support an eventual diagnostic decision. 202 referrals were submitted for adult psychiatric screening during the relevant time period for this study, and 151 completed screening. The disorders most frequently included in the referral were: ADHD combined subtype (74.26%), generalized anxiety disorder (60.89%), major depressive disorder (55.45%), social phobia (23.76%), panic disorder (20.79%), bipolar 2 disorder (15.34%), and bipolar 1 disorder (14.85%). As the study datasheet was deidentified to protect privacy during the study analyses, more exact information about the diagnostic outcomes for the entire sample is unavailable.

## Analyses

Study One’s primary analysis was an exploratory factor analysis (EFA) of participant responses to test the interrelationships among proposed ASSET-BS items to derive a parsimonious factor structure. We set a priori criteria for minimally acceptable Kaiser-Meyer Olkin score (0.70), communality with all other items (0.40), minimal factor loading within a factor (0.40), and highest acceptable cross loading onto a secondary factor (0.32). As exploratory analyses, we utilized the participant self-reported ADHD positive or negative statuses to assess preliminary discriminant/construct validity via an independent sample *t*-test for difference in means.

Study Two’s primary analysis was a confirmatory factor analysis (CFA) of the model indicated by the EFA in Study One. We then tested for possible covariate effects of demographic factors via calculating Pearson’s correlation coefficients and then, if significant, examining if the discovered covariate caused a significant difference in model fit or item loading. This test for covariate effects on factor structure was done via structural equation modeling by groups, comparing model fit and factor loading for women versus men participants.

Study Three’s primary analyses were calculations of Pearson correlation coefficients to assess convergent and divergent validity. As secondary analyses, we conducted a binary logistic regression to test for effect of gender on clinical sample ASSET-BS results, and we conducted an ROC curve analysis to assess discriminant validity strength and provide a clinical cut-off score based on optimized sensitivity and specificity. To assign ADHD positive and negative classifications to study participants, three of the authors (two doctoral-level psychologists specializing in clinical testing and assessment and one doctoral-level psychotherapist) reviewed each participant’s testing data and used the following criteria to classify the participants as ADHD positive or negative: ADHD positive status was defined as a participant that (1) had elevated T-Scores across the Brown EF/A, CAARS Self-Report, and CAARS Observer-Report Scales; (2) did not trigger any validity flags on the CAARS assessments; and, (3) the psychological assessment was ordered by a psychiatric clinician to test a hypothesized diagnosis of ADHD. If validity flags were triggered on the CAARS assessments, a participant was excluded from classification altogether.

## Results

### Study 1

Results of the EFA identified three items that did not align with factor loadings appropriately. These included *excessive talking*, *sleep quality*, and *brain fog*. Specifically, the item *excessive talking* failed to achieve a 0.40 communality, the item *sleep quality* failed to load onto a factor at a 0.40 level, and *brain fog* loaded onto both two factors over the 0.32 cut-off. After removing *excessive talking*, *sleep quality*, and *brain fog*, the EFA identified a two-factor model explaining 68.40% of variance between participants. The pattern matrix demonstrated loadings of six items onto one factor and the other four items onto a second factor. We reviewed the items belonging to each factor for conceptual fit with ADHD DSM 5 criteria. We named the first factor *Inattentive*, as the resultant items correspond conceptually with an attention deficit aspect of ADHD per criteria in the DSM 5. We named factor two *Hyperactivity and Impulsivity* due to alignment with DSM-5 criteria concerning behavioral and emotional dysregulation in an ADHD clinical presentation.

Table 1 displays the EFA-identified factor structure of the ASSET-BS. The two-factor model’s goodness of fit was calculated as χ^2^(26) = 85.69, *p* < 0.001. The factors correlated with each other significantly, *r* (155) = 0.66, *p* < 0.001, and the magnitude of the correlation coefficient indicated good convergent validity between the subscales.

**Table 1:** EFA ASSET-BS factor loadings

**Item**	**Factor 1**	**Factor 2**	**Subscale Name**
Trouble Organizing Tasks and Activities	.96		*Inattentive*
Follow-Through	.93		
Productivity	.86		
Attention Span	.66		
Misplacing Daily Items	.54		
Forgetfulness	.52		
Anxiety		.85	*Hyperactivity and Impulsivity*
Mood		.82	
Fidgetiness		.53	
Trouble Waiting Turn/General Impatience		.44	

Internal reliability for the 10-item ASSET-BS was α = 0.91. Internal reliability for the *Inattentive* subscale was α = 0.91, and the *Hyperactivity and Impulsivity* subscale’s internal reliability was α = 0.81. An independent-sample t-test found that participants who self-reported carrying a current ADHD diagnosis (*n* = 41) scored significantly higher on the ASSET-BS than participants who self-reported not carrying an ADHD diagnosis (*n* = 111), *t* (150) = 8.745, *p* < 0.001, Cohen’s *d* = 1.55.

### Study 2

Internal reliability for the 10 ASSET-BS items was α = 0.961. Little’s MCAR test indicated that any missing values were at random, χ^2^ (79) = 84.17, *p* = 0.324. A total of 0.66% (33/5910) of all possible item answers were missing. Missing item answers were imputed with a maximum likelihood method to facilitate an effective structural equation model analysis in IBM’s AMOS software package. The confirmatory factor analysis (CFA) performed via a structural equation model analysis of the two-factor item-loading model discovered in Study 1 (Theoretical Model) found strong standardized regression weights for factor loadings of all items onto their hypothesized factors. In assessing model fit, the analysis supported a finding of an acceptable-fitting model according to some indices (CFI for comparative fit, and the index PNFI indicated good model parsimony), but the RMSEA index was slightly elevated into a range indicating a degree of fit between a poor and acceptable fitting model, χ^2^ (34) = 180.19, *p* < 0.001, CFI = 0.974, RMSEA = 0.085 (90% *CI* of 0.073 and 0.098), and PNFI = 0.731. Additionally, the covariance between the latent factors (*β* = 0.96), was strong enough to raise a question of whether a one-factor model may be the optimal way to configure the items.

**Fig. 1:**
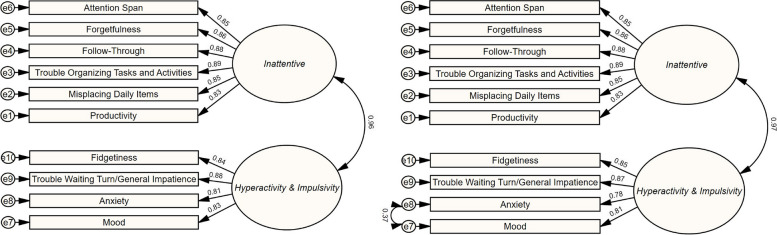
CFA via structural equation model results: tests of the theoretical model (left) and modified model (right)

Inspecting the standardized residual matrix revealed no significant measurement error was introduced by any item. The modification indices identified that if the error terms of the items *anxiety* and *mood* were allowed to covary, model fit may improve noticeably. Conceptual support for allowing these items to covary was that both items load onto the same factor, and *anxiety* and *mood* are rationally highly related in that changes in one construct could logically coincide to a high degree with a change in the other. A structural equation model analysis of an adjusted model adopting covariance between *anxiety* and *mood* (Modified Model), found improved global and comparative fit statistics over the Theoretical Model with a slight reduction in parsimony, χ^2^ (33) = 110.39, *p* < 0.001, CFI = 0.986, RMSEA = 0.063 (90% *CI* of 0.054 and 0.076) and PNFI = 0.718. Figure 1 displays the path diagrams for both the Theoretical Model and Modified Model, along with the standardized regression weights of the identified interrelationships between observed and unobserved variables.

Next, given the strong level of covariance calculated between the two latent variables, we compared a hypothesized one-factor model of the ASSET-BS to the Modified Model. The one-factor model achieved minimal differences in fit statistics in comparison to the Modified Model’s fit statistics, χ^2^ (34) = 126.37, *p* < 0.001, CFI = 0.983, RMSEA = 0.068 (90% *CI* of 0.55 and 0.081) and PNFI = 0.739. As the two-factor model was identified in Study One’s findings, had conceptual alignment with the DSM-5’s two-dimensional ADHD nomenclature, and the one-factor model failed to unambiguously improve goodness of fit, we opted to retain the two-factor model as represented in Fig. 1.

To allow for future administrations of the ASSET-BS to capture the Modified Model’s improvements in measurement accuracy, factor score coefficients were calculated and are presented in Table 2. Item-level scoring coefficients should guide scoring calculations in the following steps: first determine subscale scores via a sum of the subscale’s weighted item-level scores, multiply the subscale scores by the subscale-level scoring coefficients, and then sum the weighted subscale scores together to arrive at a total factor-weighted ASSET-BS score. The scoring coefficients are adjusted so that the overall ASSET-BS factor score is a continuous integer greater than or equal to 1 and lesser than or equal to 6. A paper version of this scale including the factor-weighted scoring calculation is reproduced in Supplement 1: Appendix A.

**Table 2:** Factor score weights

**Item**	**Subscale**	**Factor Score Weight**	**Subscale Weight**	**Item-Level Coefficient for Subscale Scoring**	**Subscale-Level Coefficient for Total Scale Scoring**
Fidgetiness	*Hyperactivity and Impulsivity*	.131	.427	.31	.36
Waiting Turn		.154		.36	
Anxiety		.059		.13	
Mood		.083		.19	
Productivity	*Inattentive*	.098	.758	.13	.64
Follow Through		.144		.19	
Forgetfulness		.131		.17	
Attention Span		.119		.16	
Misplacing Daily Items		.117		.15	
Trouble Organizing Tasks and Activities		.149		.20	

#### Covariate analysis

The demographic variables were re-coded as categorical (race, gender, geographic area of the United States, and urban or rural) or continuous (age and level of education). Of the possible covariate variables, initial correlation (continuous variables) and ANOVA (categorical variables) analyses, found that gender may possibly impact ASSET-BS scores significantly with a non-negligible effect size, *F* (1) = 19.10, *p* < 0.001, Cohen’s *d* = 0.37. As non-cisgender participants made up a very small portion of the sample, only scores from participants who identified as cis-gender were entered into an ANOVA to identify differences in mean ASSET-BS scores based on gender (cis-women: *M* = 3.23, *SD* = 1.52; cis-men: *M* = 3.78, *SD* = 1.47).

To test whether gender affected the CFA in terms of either factor loading or model fit, we compared CFA results via separate structural equation model analyses, splitting the sample by gender groups (cis-gender male and cis-gender female). For the two groups analyzed, all items loaded onto their hypothesized latent variables without noticeable variation from the CFA tests of the Theoretical Model and Modified Model. The model’s fit statistics for the cis-gender women group were χ^2^ (33) = 105.35, *p* < 0.001, CFI = 0.976, RMSEA = 0.084 (90% *CI* of 0.66 to 0.102) and PNFI = 0.708. The model’s fit statistics for the cis-gender men group were χ^2^ (33) = 68.33, *p* < 0.001, CFI = 0.986, RMSEA = 0.063 (90% *CI* of 0.41 and 0.084) and PNFI = 0.714. For the cis-gender women group, covarying the error terms of the executive functioning items *productivity* and *misplacing daily items* improved model fit χ^2^ (32) = 87.63, *p* < 0.001, CFI = 0.981, RMSEA = 0.075 (90% *CI* of 0.56 to 0.094) and PNFI = 0.691, but had negligible effect on model fit when applied to the cis-gender men group, χ^2^ (32) = 65.71, *p* < 0.001, CFI = 0.987, RMSEA = 0.062 (90% *CI* of 0.41 to 0.084) and PNFI = 0.693. Recalculating scores for cis-gender women participants using adjusted factor weights (reproduced in Supplement 2: Appendix B) to reflect the allowance of covariance between *productivity* and *misplacing daily items* negligibly reduced the effect size of gender on ASSET-BS scores, falling from Cohen’s *d* = 0.37 to Cohen’s *d* = 0.35.

### Study 3

Internal reliability of the ten ASSET-BS items was ⍺ = 0.899. In this sample, Gender was found to have a small but significant correlation with ASSET-BS scores *r* (143) = 0.232, *p* = 0.005. The cis-gender men’s mean ASSET-BS score was 3.47, *SD* = 1.23 and the cis-gender women’s mean ASSET-BS score was 4.04, *SD* = 1.10. A binary logistic regression analysis found that gender accounted for 5.2% of the variance between participants in ASSET-BS scores, Cox & Snell *R*^2^ = 0.052, χ^2^ (1) = 7.07, *p* = 0.006. Adjusting the women’s ASSET-BS scores by using Study 2’s alternative factor weighting for women had no impact on the effect size of gender on difference in mean ASSET-BS scores.

#### Discriminant validity

Using the classification criteria outlined in the Analyses section, the three-author team classified 30 participants as ADHD positive, 93 as ADHD negative, and 12 participants were excluded from discriminant validity analyses due to internal-validity flags contained in their CAARS results reports.

Table 3 displays the descriptive statistics of the ADHD positive and negative groups in relation to the ADHD-relevant measures that were administered. In this sample, the ADHD-positive group self-reported a strikingly high level of symptom severity compared to more conservative estimates assessed by the CAARS-observer report scales; however, the ADHD positive group’s scores had lower standard deviation values than the ADHD negative group.

**Table 3:** ADHD positive and negative participant descriptive statistics

**Scale**	**ADHD Positive Mean** ***n* = 30**	**ADHD Positive Standard Deviation**	**ADHD Negative Mean** ***n* = 93**	**ADHD Negative Standard Deviation**
Brown EF/A Index T-Score	82.87	5.36	62.24	10.47
CAARS Self-Report Index T-Score	72.20	6.87	54.97	9.02
CAARS Self-Report Symptoms T-Score	85.13	9.50	57.80	13.50
CAARS Observer-Report Index T-Score	68.96	10.52	54.97	9.02
CAARS Observer-Report Symptoms T-Score	68.32	11.90	52.00	9.99
ASSET-BS Factor Score	4.91	0.59	3.43	1.06

**Fig. 2:**
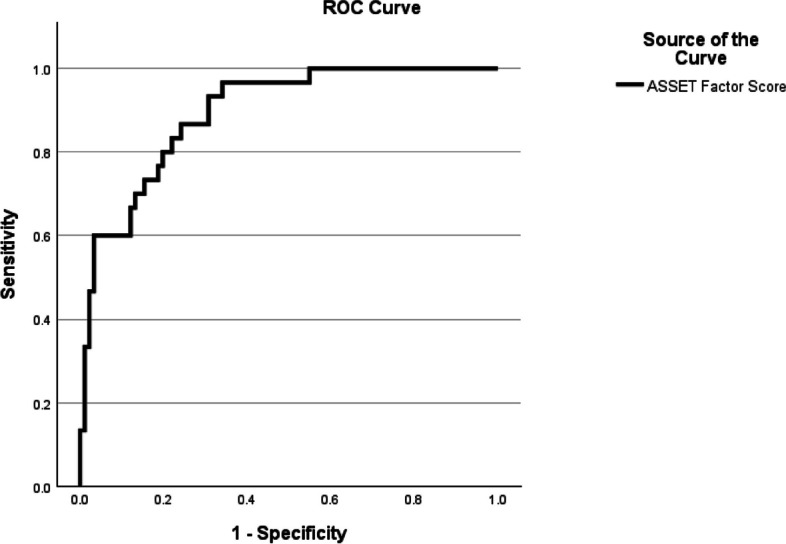
ROC curve graph

The receiver operating characteristic (ROC) curve analysis of the ASSET-BS calculated an AUC coefficient of 0.895, (95% *CI* = 0.835 to *0.9*54), and a Gini index coefficient of 0.789. An ASSET-BS factor score of 4.04 achieved 96.7% sensitivity and 65.9% specificity, positive predictive value (PPV) = 47.54%, negative predictive value (NPV) = 98.39%. To better balance sensitivity and specificity, a score of 4.40 achieved 80.0% sensitivity and 80.2% specificity, PPV = 57.14%, NPV = 92.60%. Figure 2 displays the graph of the ROC curve line.

#### Convergent and divergent validity

The ASSET-BS strongly correlated with published self-report measures of ADHD, specifically the Brown EF/A Index Score *r* (131) = 0.76, *p* < 0.001, the CAARS Self-Report ADHD Index, *r* (131) = 0.71, *p* < 0.001, and the CAARS Self-Report DSM-5 ADHD Symptoms Index, *r* (131) = 0.68, *p* < 0.001. The ASSET-BS moderately correlated with the CAARS Observer-Report ADHD Index (*r* (104) = 0.49, *p* < 0.001), as well as the CAARS Observer-Report ADHD Symptoms Score, *r* (104) = 0.45, *p* < 0.001. The correlation coefficients of the relationships between the ASSET-BS and the CAARS Observer-Report scores were similar in magnitude to the correlation coefficients describing the relationship between the CAARS Self-Report ADHD Index and the CAARS Observer-Report ADHD Index, *r* (107) = 0.55, *p* < 0.001, and the CAARS Observer-Report DSM 5 Symptoms Score, *r* (107) = 0.51, *p* < 0.001.

To assess divergent validity, correlations between the ASSET-BS and measures for sleepiness, depression and OCD were calculated. The ASSET-BS moderately correlated with the PHQ 9, *r* (143) = 0.57, *p* < 0.001. It weakly correlated with the Epworth Sleepiness Scale (ESS), *r* (143) = 0.22, *p* = 0.009, and had no significant correlation with the Dimensions of Obsessions and Compulsions Scale (DOCS), *r* (83) = 0.111, *p* = 0.319.

## Discussion

Results from the three studies indicate that the ASSET-BS is a valid and reliable ADHD screener with a focus on clinical use. To understand how the ASSET-BS fits within the current set of ADHD scales developed for clinical use, the Adult ADHD Quality of Life Scale (AAQOL) [34] and the ADHD-Rating Scales (ADHD-RS) [35] serve as interesting comparison scales. The AAQol and the ASSET-BS both capture ADHD symptom severity in relation to impact on daily life functioning. The AAQoL has been found to detect changes in life functioning in response to ADHD treatment [36]. A study evaluating the ASSET-BS in measuring sensitivity to treatment is currently in preparation. Considering that the AAQoL has 29-items, the 10-item ASSET-BS may be a more efficient and accessible option for routine clinical use, especially if it likewise can prove to capture changes in symptom severity due to treatment. The clinician-rated ADHD-RS is also used as a tool to identify ADHD cases and measure treatment progress in clinical contexts. Like the ASSET-BS, the ADHD-RS has two subscales, *Inattention* and *Hyperactivity*, aligned with diagnostic criteria [34]. However, the ADHD-RS is an instrument designed to be administered by a clinician in a structured interview, and thus routine administration of the ADHD-RS can require significant expenditure of time and relies heavily on clinician expertise.

We found encouraging results in support of convergent validity, as the ASSET-BS strongly correlated with the Brown EF/A and the CAARS Self Report Index and the CAARS Self Report DSM-5 Symptoms Subscale. For divergent validity, correlations with the ESS and the DOCS were low, showing that the ASSET-BS measurements are not overly impacted by divergent constructs from ADHD. However, the moderate correlation with the PHQ-9 shows that depressive symptoms may impact ASSET-BS scoring, and clinicians should likewise interview for depression signs and symptoms when reviewing an elevated ASSET-BS score to ensure the score is reflective of ADHD and not a major depressive disorder.

### Limitations

ASSET-BS scores are impacted to a small degree by gender identity. However, the impact of gender on ASSET-BS results requires further exploration due to differing results between Study 2 and Study 3. In Study 2, the general population study, the mean male score was elevated above the mean female score, whereas in Study 3, the clinical sample study, the female mean score was elevated above the male mean score. Because ADHD is a neurodevelopmental disorder with multiple biopsychosocial elements at play in its causation and presentation, it is unsurprising that gender identity would have measurable effects on symptomatology [10]. We encourage clinicians when using any scale in the evaluation of ADHD to complement scale scores with an interview of the patient focused on how quantitative results complement or contrast with lived experiences and the patient’s social history and context. Moreover, the current study did not consider variations in screening scale performance across different clinical subtypes within the sample in Study 3. A more in-depth consideration of how scores vary across different clinical populations is a fruitful avenue for future research.

A key caution is that an ADHD diagnosis cannot be supported based on an ASSET-BS result alone. The ASSET-BS is intended to aid in screening for ADHD via measuring how severely ADHD symptoms are impacting daily life functioning. If a patient’s ASSET-BS score is elevated at or above the clinical cut-off score, further clinical assessment procedures should follow to establish whether or not ADHD is a consistent and parsimonious explanation of the patient’s clinical presentation, and to assess possible malingering or drug-seeking behavior [37]. Further, while the ASSET-BS items were identified from a set of items constructed by psychiatric practitioners in the course of treatment of ADHD, it is yet to be established if repeat administrations of the ASSET-BS can reliably and accurately evaluate ADHD treatment efficacy.

Generalizability limitations exist for the clinical sample study results. The clinical sample was homogenous in terms of race/ethnicity and so future studies should evaluate the ASSET-BS’ clinical performance within diverse clinical populations. Study Two, the general population study, did not find significant differences in ASSET-BS scores by race/ethnicity. Even so, we cannot rule out the possibility of race/ethnicity, or a variety of other demographic variables, i.e. nationality, religion, region, social economic status, etc., being significant covariates within clinical populations. Thus, a key clinical use caution is to ensure that ASSET-BS results are considered in the context of intersecting multicultural factors possibly impacting symptoms.

## Conclusion

The ASSET-BS is designed to be used at intake in an outpatient psychiatric setting to conduct an effective screening for probable ADHD positive cases. These results establish that the ASSET-BS is a valid tool for clinical screening use. Study One identified a 10-item scale, with two factors, that combined explained 68.40% of the variance in participant answers. Study Two confirmed the two-factor model identified in Study One in a large sample whose demographic characteristics were representative of the U.S.A. general population. Study Two also identified measurement improvements through incorporating into scoring expected covariance between two items, and provided factor scoring coefficients so that future ASSET-BS scoring can be consistent with the improved measurement model. Study Three established that the ASSET-BS, as scored in accordance with the factor scoring identified in Study Two, had good convergent validity and acceptable divergent validity. Study Three’s results also demonstrated good discriminant validity for use within a clinical sample, with 80.0% sensitivity and 80.2% specificity found at a cut-off factor score value of 4.40. All three studies’ results supported high internal reliability with α values in Studies One, Two and Three ranging from α = 0.899 to α = 0.961. Future research should investigate the ASSET-BS’ ability to measure change due to psychiatric treatment, evaluate test-retest reliability, how the ASSET-BS performs in more diverse clinical samples, and further explore the effects of gender on scoring. Lastly, as a clinical use caution, to counter any effects of gender or other demographic variables on ASSET-BS scores, we strongly advise considering ASSET-BS results in the context of a biopsychosocial interview to understand how a multitude of biopsychosocial factors, such as gender norms, roles, and expectations may impact item-level scores.

### Supplementary Information

**Additional file 1: Appendix A.** ADHD Symptom and Side Effect Tracking - Baseline Scale.

**Additional file 2: Appendix B.** Alternative Factor Score Calculation for Women.

## Data Availability

The de-identified data and materials supporting the findings of this study are available upon reasonable request made to the corresponding author.
