# Supplementary material for: Development and validation of the ADHD Symptom and Side Effect Tracking - Baseline Scale (ASSET-BS): a novel short screening measure for ADHD in clinical populations
Source: BMC Psychiatry. 2023 Nov 6;23:806. doi: 10.1186/s12888-023-05295-6 (PMC10629079; doi:10.1186/s12888-023-05295-6)
Supplement: Supplementary file 1 — Additional file 1: Appendix A. ADHD Symptom and Side Effect Tracking - Baseline Scale. [file 12888_2023_5295_MOESM1_ESM.docx]

**Supplement 1: Appendix A**

*ADHD Symptom and Side Effect Tracking - Baseline Scale*

**Instructions**

Please indicate on a scale of 1 to 6 how strongly *problems* with each of the following impacts your daily life functioning. Daily life functioning includes activities such as recreation, relationships, work, family, household chores, etc.

*Scale:*  **1** = No problem present, **2** = Noticeable but no impact, **3** = Mild impact,

**4** = Moderate impact, **5** = Strong impact, **6** = Severe impact

*Inattentive Subscale*

Attention span ____ x 0.16 = ____

Forgetfulness ____ x 0.17 = ____+

Follow-through ____ x 0.19 = ____+

Trouble organizing

Tasks and activities ____ x 0.20 = ____+

Misplacing daily

Items ____ x 0.15 = ____+

Productivity ____ x 0.13 = ____+

**Inattentive Score: ____**

*Hyperactivity and Impulsivity Subscale*

Fidgetiness ____ x 0.31 = ____

Trouble waiting turn/

General impatience ____ x 0.36 = ____+

Anxiety ____ x 0.13 = ____+

Mood ____ x 0.19 = ____+

**Hyperactivity and Impulsivity Score: ____**

*Total Score*

Inattentive Score ____ x 0.64 = ____

Hyperactivity and Impulsivity Score ____ x 0.36 = ____+

**ASSET-BS Total Score: ____**

**Side Effect Questions**

(For use with the ASSET-BS questions during follow-up clinic visits)

Please indicate how frequently you have experienced each of the following over the past two weeks.

*Scale:*  **1** = Never, **2** = Rarely, **3** = Occasionally,

**4** = Often, **5** = Always

*Items:* Insomnia

Generalized pain

Fatigue

Dry mouth

Poor appetite

Food binges

Tics

Anger

Suspiciousness

Restless legs

End of dose crash

Return of symptoms as dose wears off

Unwanted changes in weight*

**For this item use the guide below:*

Always = A very concerning/surprising amount of weight change,

Often = A concerning amount/worrisome level of unwanted weight change,

Occasionally = A noticeable amount of unwanted weight change but not to the point of causing worry,

Rarely = A slight/negligible amount of unwanted weight change,

Never = No unwanted weight change.
