# Supplementary material for: Development and validation of the ADHD Symptom and Side Effect Tracking - Baseline Scale (ASSET-BS): a novel short screening measure for ADHD in clinical populations
Source: BMC Psychiatry. 2023 Nov 6;23:806. doi: 10.1186/s12888-023-05295-6 (PMC10629079; doi:10.1186/s12888-023-05295-6)
Supplement: Supplementary file 2 — Additional file 2: Appendix B. Alternative Factor Score Calculation for Women. [file 12888_2023_5295_MOESM2_ESM.docx]

**Supplement 2: Appendix B**

*Alternative Factor Score Calculation for Women*

| **Item** | **Subscale** | **Factor Score Weight** | **Subscale Weight** | **Coefficient for Subscale Scoring** | **Coefficient for Total Scale Scoring** |
| --- | --- | --- | --- | --- | --- |
| Fidgetiness | *Hyperactivity and Impulsivity* | .138 | .490 | .28 | .38 |
| Waiting Turn |  | .175 |  | .36 |  |
| Anxiety |  | .063 |  | .13 |  |
| Mood |  | .114 |  | .23 |  |
| Productivity | *Inattentive* | .142 | .792 | .18 | .62 |
| Follow Through |  | .139 |  | .18 |  |
| Forgetfulness |  | .130 |  | .16 |  |
| Attention Span |  | .102 |  | .13 |  |
| Misplacing Daily Items |  | .148 |  | .19 |  |
| Trouble Organizing Tasks and Activities |  | .131 |  | .16 |  |
|  |  |  |  |  |  |
